# Short and medium-term effects of a multicomponent physical exercise program with a Mediterranean diet on bone mineral density, gait, balance, and fall risk for patients with Alzheimer disease: Randomized controlled clinical trial study protocol

**DOI:** 10.1097/MD.0000000000022385

**Published:** 2020-09-18

**Authors:** Ana Silvia Puente-González, Felipe Sánchez-González, Juan Elicio Hernández-Xumet, María Carmen Sánchez-Sánchez, Fausto José Barbero-Iglesias, Roberto Méndez-Sánchez

**Affiliations:** a Department of Nursing and Physical Therapy. University of Salamanca, Salamanca, Spain; b Institute of Biomedical Research of Salamanca (IBSAL), Salamanca, Spain; c Department of Physical Medicine and Pharmacology, University of La Laguna, Santa Cruz de Tenerife, Spain.

**Keywords:** Alzheimer disease, bone density, exercise, falls, gait, Mediterranean diet

## Abstract

**Introduction:**

Reduced bone mineral density and increased risk of falls are related with Alzheimer disease, and these increase likelihood of bone osteoporotic fractures causing serious complications such as disability, fear of falling, loss autonomy, decreased quality of life, and anticipated mortality in elderly patients. Gait and balance disturb are 2 factors to favor falls in elderly, and in patients with cognitive impairment, the risk of falls increases to double. Exercise and Mediterranean diet produce beneficial effects for aging, cognitive decline, and are widely recommended to reduce the effects of osteoporosis, fall risk, and related fragility fractures. The primary objective of this study is to evaluate the short and medium-term effects during 6 months, of a multicomponent physical exercise program with a Mediterranean diet on bone mineral density, fall risk, balance, and gait by a controlled clinical trial in patients with Alzheimer disease.

**Methods:**

The study is a 6-month, randomized controlled parallel-group, single-blinded clinical trial. Institutionalized patients with Alzheimer disease will be included. The intervention group will perform a multicomponent physical exercise program in reduced groups, with a frequency of 3 sessions per week, associated with a Mediterranean diet. This program includes strength, balance, and aerobic resistance exercises, and in the main part of the session, also ludic exercises to improve agility, coordination, and balance. The control group will receive usual care. The outcomes to assess are the change of physical functions, such as gait and balance, and the change of bone mineral density by calcaneal quantitative ultrasound, during the study follow-up at 1, 3, and 6 months. This clinical trial will generate more and new evidence on the effects of a multicomponent physical exercise program and Mediterranean diet in patients with Alzheimer disease on risk of falls and osteoporotic fractures, the relation of these with bone mineral density, gait and balance, and the correlations between them.

**Ethics and dissemination:**

This study protocol has been approved by the Ethics Committee of the University of Salamanca. The results will be published in peer-reviewed journals and disseminated in national and international conferences, to the participants and their families, and the general public through the associations of people with AD.

**Trial registration ID:**

ClínicalTrials.gov ID: NCT04439097.

## 1 Introduction

Alzheimer disease (AD) is a neurodegenerative disease, the leading cause of dementia worldwide. AD is associated with the aging process,^[1,2]^ and is showing an increasing prevalence in occidental countries, such as other chronic diseases associated with increased life expectancy.^[3]^ If dementia prevalence data are maintained, from the 2015 data, there would be an approximate 300% increase by 2050, with around 130 million patients with dementia.^[4]^ According to an estimate by Alzheimer's Disease International, the global cost of the disease in 2015 amounted to 818 billion (USD$), a 35% more than in 2010, and is expected to increase to 2 trillion (USD$) by 2030.^[5]^

Cognitive impairment and frailty are related and share pathophysiological bases and some results such as falls, fractures, disability, even mortality,^[6,7]^ and both clinical processes also share low physical activity and gait disturbances.^[7]^ In the same way, bone fragility is a clinical comorbidity in AD,^[8]^ and low bone mineral density (BMD) occurs at twice the rate in AD patients as in healthy elderly adults.^[9]^ A long-term study, with a 3-year follow-up, suggested that there is a significant association between cognitive impairment and low BMD.^[8,10]^ It even showed that older women with reduced BMD are at a higher risk of cognitive decline, in accordance with Chang et al,^[11]^ who evidenced an increased risk of dementia in subjects with diagnosis of osteoporosis or osteoporotic fractures in Asian populations. In addition, it is known that low bone mass conditions weaken the skeleton and increase likelihood of bone fractures.^[12–14]^

Falls are directly related to bone fractures, causing significant complications such as increased risk of disability, decreased quality of life, fear of falling, loss of autonomy, and anticipated mortality in elderly patients.^[12–15]^

Changes during aging in sensor-motor and vestibular systems influence mobility and balance, increasing the risk of falls.^[16,17]^ This has also been demonstrated in AD patients, but this association is not well understood yet. Furthermore, it is known that patients over 65 with dementia have a 2-fold increased risk of falls, compared with no demented elderly.^[17–20]^

Cognitive deficit in elderly patients with AD is associated with gait and balance disorders,^[21–25]^ and influences executive functions, attention, and visuospatial perception and enhances the risk of falls.^[25–29]^ Yoon et al^[21]^ suggest that impairment in balance and mobility begins during the subjective cognitive decline stage, especially among the most vulnerable populations of women and APOEε4 carriers; therefore, we can consider that the impairment in balance and mobility could represent a predictive surrogate marker of cognitive decline.^[21]^

Gait alterations appear in 50% of AD patients 3 years after the diagnosis of AD, and among these, 33% lose their capacity to walk. The prevalence of gait and balance alterations ranges from 9% to 52% depending upon the tool of assessment.^[20,22,30]^

There are many tests used to assess gait and balance in elderly, cognitive deficit, or AD patients, Tinetti Performance Oriented Mobility Assessment (POMA-T), timed up and go test (TUG), 1-leg balance test (OLB), and functional reach test (FR) being among the most used.^[16,20,21,30–34]^ For this reason, they were selected for use in our study.

Physical activity and exercise have been shown that can delay the progress of AD in an effective cost and sustainable manner.^[35]^ Currently, we know that exercise achieves improvement in cognition of elderly with AD,^[35,36]^ and have other beneficial effects improving physical function, increasing autonomy, and quality of life in patients with dementia.^[3,30,31]^ The studies showed evidence with different factors such as type of exercise, frequency, intensity, time, and follow-up of the intervention, participants, etc.^[30,35]^ Besides, pharmaceutical treatments fail to address the cause of AD, and only a few symptomatic treatments are currently available, providing modest improvements to patients’ livelihood, and normally with side effects that may include nausea, dizziness, and weight loss.^[2,37,38]^ Exercise has shown even more positive results in terms of mortality from different diseases and chronic conditions than poly-pills for cardiovascular prevention.^[39]^

Exercise can contribute to mitigate cognitive decline and AD because among some of its effects are to improve cardiovascular health and increase muscle mass, reduce oxidative stress and inflammation, and stimulate hippocampal plasticity,^[2,40]^ also, taking into account the possible effect on the appearance or delay chronic diseases,^[2,41]^ and thus, improve their quality of life and significantly reducing the global burden of AD representing a main nonpharmacological lifestyle intervention.^[2,42,43]^

Along with exercise, diet is another major factor in maintaining a lifestyle that has been shown to have positive effects on the risk of dementia and cognitive impairment.^[44–46]^ The Mediterranean diet (MeDi) has shown as a promise dietary pattern in recent research, decreasing dementia risk, through altering cardiovascular risk factors and lower levels of neuropathology,^[44]^ and fracture risk through improving bone and muscle health status.^[47]^ In general, the literature support that MeDi is one of the best diet model to maintain health, with a lower incidence of frailty or disability in old age.^[48]^ The MeDi is predominantly plant-based, with a high intake of vegetables, fruits, nuts, and legumes, moderately high intake of fish, low intake of red meat, and includes extra virgin olive oil.^[49]^

The American College of Sports Medicine recommends 150 minutes of moderate or 75 minutes of vigorous physical activity per week, preferably divided into 3 to 5 sessions, and muscle-strengthening activities 2 or more days per week, with a recommendation of 3/2 ratio of aerobic and strength exercise per week.^[2,50,51]^

A multicomponent physical exercise program (MPEP) is possibly the best intervention option to obtain greater beneficial effects in older people.^[7,52–54]^

Strength training, resistance, and balance, combined or separately, have shown beneficial effects on several symptoms or factors associated with AD. So, we can consider exercise as a useful tool to improve functional capacities as mobility, gait, balance and strength, executive functions, and thus decrease falls.^[7,24,36,52–58]^ Moreover, we must consider that exercise or planned physical activity is a helpful plan for maintaining optimal bone health regulating bone metabolism,^[41,59,60]^ although its mechanism to improve bone health is not exactly clear yet. However, it has been accepted that increasing muscle mass and mechanical stress in bones results in increasing or preventing the loss of BMD.^[41,61,62]^ Evidence has shown that regular exercise with moderate intensity would decrease bone resorption and increase bone mass in both healthy and pathologic subjects.^[63]^

For the above reasons, a MPEP was designed in elderly people diagnosed with AD through a group intervention that will be described later. Group interventions have been shown to have high therapeutic potential.^[64,65]^

### 1.1 General and specific objectives

The general objective of this MPEP associated with a MeDi is to decrease the risk of falls and fractures through the improvement of the bone health and physical functions of people with AD temporarily admitted to a Center for people with Alzheimer's Disease and other Dementias (Center-AD). Specific objectives include evaluating the effect and the changes of the MPEP and a MeDi on bone mass, physical functions, and their relationship to fall risk. Therefore, the aim of this study is to evaluate the short and medium-term effects, during 6 months, of a MPEP and MeDi on BMD, fall risk, balance, and gait by a randomized controlled clinical trial in patients with AD.

## 2 Methods

### 2.1 Study design

This study is a randomized, controlled, parallel-group, single-blinded clinical trial. The protocol of the clinical trial received approval from the Ethics Committee of University of Salamanca, and shall be carried out in accordance with the Declaration of Helsinki. The informed consent is written and accessible in Spanish with the approved protocol. The clinical trial was registered in ClínicalTrials.gov ID: NCT04439097.

The study protocol conforms to the SPIRIT 2013 Statement (Standard Protocol Recommendations for Interventional Trials),^[66]^ and the Clinical Trial conforms to the CONSORT 2010 Statement (Consolidated Standards of Reporting Trials).^[67]^

### 2.2 Recruitment/enrollment and allocating

**Figure 1:**
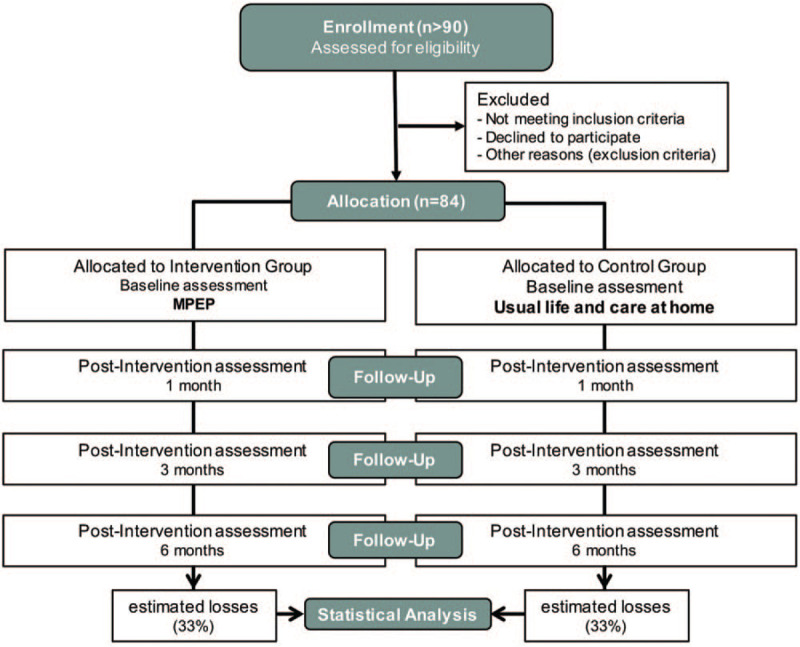
Flowchart of participants in the study.

Patients who request for admission to the Center-AD, and who meet the initial selection criteria will be invited to participate in the study. Patients and their families will receive oral and written information by the physiotherapist of the Center-AD, who will apply the intervention, and, after signing the informed consent (by the patient or the legal guardian), the patients will be cited to make the baseline assessment with a multidisciplinary evaluator team. They will be randomized and assigned to the study groups. The patients in the intervention group will be admitted, and in the control group, they will be placed on a waiting list for admission once their participation in the study has ended. The randomization will be undertaken by the physiotherapist and an independent member of the Direction of the Center, with no other involvement in the trial, by using a computerized randomization system (randomized.com). Into our research line, the data obtained in a pilot study and a preliminary clinical trial have helped to improve the design of our current study. See the flowchart in Figure 1.

### 2.3 Blinding

The multidisciplinary care team, with a neurologist, a neuropsychologist, and a physiotherapist, will perform all evaluations without knowing if the patients were enrolled and the group to which the patients have been assigned, and they will not have a relationship with the physiotherapist who applies the intervention. An independent researcher, who will not know the identification of the groups, will carry out the statistical analysis.

### 2.4 Inclusion criteria

Patients are included in the study if they meet the following inclusion criteria:

- Patients of both sexes older than 50 years;
- Have a diagnosis of AD;
- Present mild or moderate cognitive impairment [Mini-mental State Examination (MMSE) score between 11 and 23 points included]^[68]^;
- Acceptance to participate in the study (enrollment in the study and signing of informed consent).

### 2.5 Exclusion criteria

Patients are excluded if they present the following exclusion criteria:

- Other associated pathologies that do not allow physical exercise due to having severe functional disability or being insecure (neurological diseases, severe cardiorespiratory pathology, etc);
- Impossibility to carry out the evaluation tests;
- Falls and other incidents or adverse events with severe consequences that cause disability and/or that do not allow the intervention to continue;
- Attendance at the MPEP is less than 75% in the total of the sessions between assessments. (Criterion applied to each period between assessments). Adherence control by the physiotherapist;
- Not performing the MPEP sessions for 2 or more consecutive weeks;
- Participate in another intervention program outside of this study, especially an exercise program (important intervention bias for the control group).

### 2.6 Interventions

#### 2.6.1 Intervention group

Patients allocated to the intervention group will perform a MPEP during 6 months, with a frequency of 3 sessions per week, and approximately 45 to 50 minutes of duration each session, in addition to having to a MeDi.

The diet will follow the recommendations of a nutritionist in accordance with Davis et al^[49]^ and the main recommendations are ≥5 servings of vegetables/day; ≥3 servings of fruit/day; ≥3 serves of fish or seafood/week; ≥3 servings of legumes/week; ≥3 servings of nuts/week; ≥5 servings of bread or cereals/day; preferentially consume white meat, instead of red meat; 2 to 4 servings of poultry/week and ≤1 serving of red meat/week; 2 to 4 servings of eggs/week; and using olive oil as the main oil for cooking and dressing.

The design of the program is based on previous studies and recommendations where it is said that the intervention with different types of exercises has better benefits in elderly and frail people, as the European VIVIFRAIL program applied in frail community elders with cognitive decline.^[7,52,69–71]^ In other studies, for patients with osteoporosis, it is recommended to work primarily with progressive resistance training for all major muscle groups and balance exercises and it is not recommended to work with aerobic exercises.^[72,73]^ In addition, we have considered some proposed guidelines for the treatment of postmenopausal and senile osteoporosis,^[74]^ for the prevention of falls^[57,71]^ and we have also revised the guidelines from the position stand on Exercise and Physical Activity for Older Adults of the American College of Sports Medicine.^[75]^

The participation of the patients in the MPEP will be carried out in small groups of 5 to 8 people, which enables to have the benefits of collective work and, at the same time, allows the physiotherapist to supervise and instruct each patient at all times in a safe way. The distribution of patients in these small groups will be made homogeneously based on functional and cognitive impairment tests in the baseline assessment.

**Figure 2:**
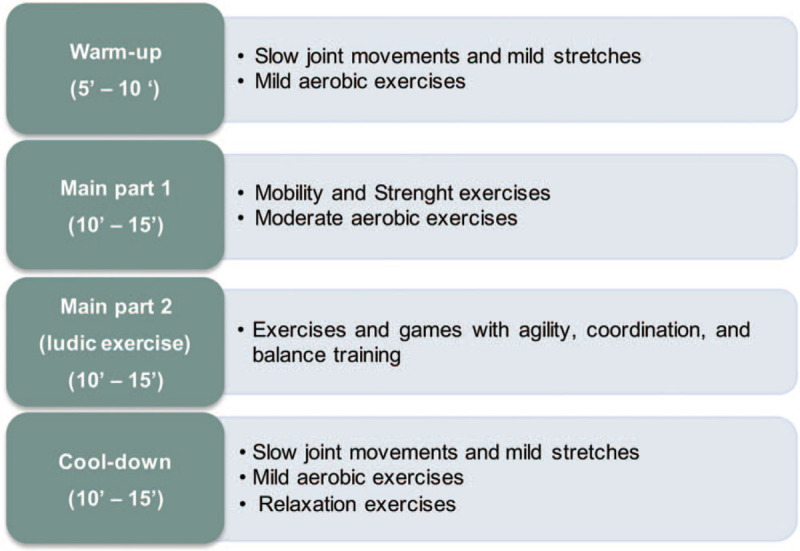
Structure of sessions in the multicomponent physical exercise program.

The structure of the sessions has been defined according to the recommendations of the American College of Sports Medicine,^[50]^ with 3 different parts: an initial warm-up, a main part, and a final cool-down and relaxation, as is shown in Figure 2. In this basic structure, traditional exercises of mobility, strength, balance, and coordination are included with the main objective of improving functional capacities. But, besides, games and some activities are also included with the aim, not only of improving the functional capacity but also of working the cognitive functions to reinforce the global effects of the MPEP.^[36]^ During the sessions, the physiotherapist will constantly promote social participation among the participants.

As general premises, all exercises will be performed following a progression in dosage, intensity, and difficulty. This progression will be especially important in the exercises of strength, balance, and aerobic resistance, as we intend that initially there is an adaptation of the patient to the exercise, but later we will try to make that they always be a challenge to the patients.

During the sessions, the physiotherapist will give simple instructions to do the exercises, performing them before and simultaneously with the patients to help them to do the exercises by imitation, and giving them positive reinforcements constantly. The physiotherapist should try to ensure that the patients perform all the exercises as correctly as possible with the expected dose and intensity, although it is necessary to be careful and always consider possible exercises adaptations to the functional and cognitive state of the patient in a safe way.

The basic session of the MPEP will be structured into 3 parts (Fig. 2):

(1) Warm-up: The sessions begin with an initial warm-up from 5 to 10 minutes.

The patients will start with slow joint movements and mild stretches of main muscle groups. Depending on the functional capacity of the patients in each group, they can start seated on a chair, and later perform the exercises in standing position. The upper quadrant (upper limbs, shoulder girdle, head, and neck) could be worked in a sitting or standing position. The lower quadrant (lower limbs and trunk) will be worked in a standing position fundamentally, although sometimes it will also be possible working in a sitting position. Subsequently, mild aerobic training will begin where there are combined upper limbs movements, walking, pedal sitting, etc, always depending on the functional capacity of the patients and progressively increasing the intensity. Mild aerobic work can be alternated with mobility and stretching exercises.

(a) Main Part: The main part of the session will be divided into 2 with an approximate duration of 10 to 15 minutes each part.
(b) Main Part 1: Some traditional exercises will be done to work on joint mobility but, fundamentally, strength exercises will be performed on the main muscle groups, alternating the work of the upper quadrant and lower quadrant muscles. In these strength exercises, the progression in the dose, intensity and difficulty should also be applied.

In addition, this part of the session includes moderate aerobic exercises that will be gradually increased, with different exercises as upper and lower limbs movements, walking, running, pedal sitting, indoor cycling, etc.

(a) Main Part 2 (ludic exercises): This part of the session will include different games, mainly group games, to improve agility, coordination and balance. Traditional and simple games, known to patients, will be carried out, with rules that are easy to understand and execute, and that allow to achieve the stated objectives.

For the main part, an extensive rehabilitation and fitness equipment will be used to perform the games. Exercises and games will be performed in different positions and surfaces and will be accompanied by a dual task activity (physical and cognitive) that also promote the social participation of patients.^[76,77]^

(1) Cool-down: In this final part of the session, mild aerobic exercises will be performed to progressively decrease cardiopulmonary and muscular activity. In static, standing, or sitting, patients will perform slow joint movements and mild stretches. Finally, preferably in a sitting position, patients will be instructed to perform relaxation exercises, trying to control breathing.

#### 2.6.2 Control group

Participants allocated to the control group will receive usual care and continue with their life normally, without participating in a standardized exercise program, and with the possibility to receive physical rehabilitation when needed. They will be instructed to maintain their current physical activity level, and will have to carry out the evaluations provided for in the study at 1, 3, and 6 months, with a periodic telephone contact. When the control patients are admitted to the Center-AD, they will have the opportunity to participate in the MPEP as well as the intervention group.

### 2.7 Outcome variables

In the baseline assessment, all variables will be measured including the sociodemographic variables. Later, all outcome variables will be measured at 1, 3, and 6 months (Fig. 1) and will be codified by the physiotherapist who will conduct the intervention. A summary of outcome variables in summarized in Table 1.

**Table 1:**
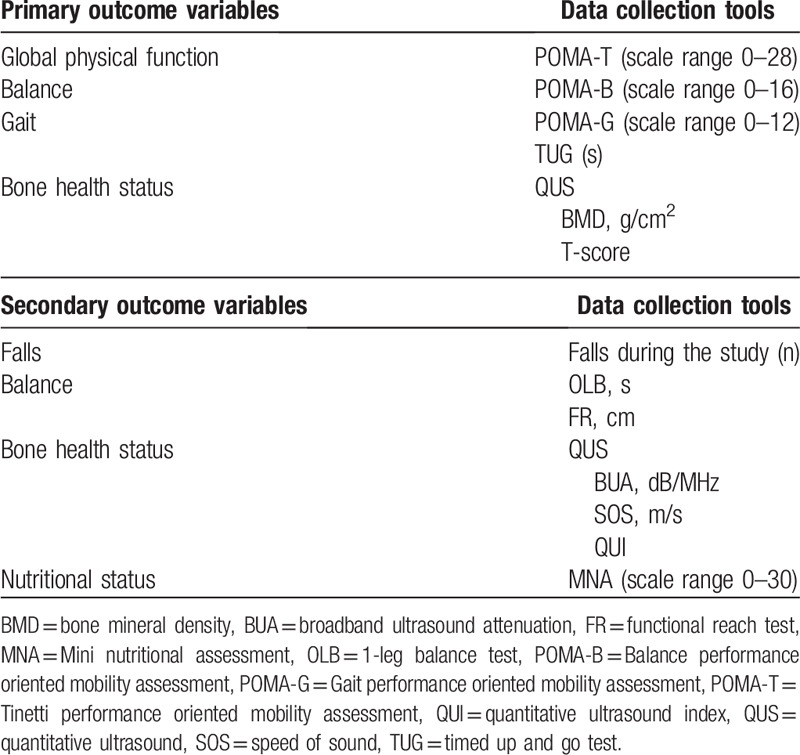
Summary of primary and secondary outcome variables assessed at baseline, 1, 3, and 6 months’ follow-up.

A pilot study and a preliminary study were undertaken to establish the appropriateness of assessment tools and the standardization of procedures.

Personal and sociodemographic variables are as follows:

- Age (years);
- Sex (male or female);
- Weight (kg); Height (m); body mass index (BMI) (kg/m^2^);
- Study level (no studies, basic education, secondary school, or superior studies);
- Marital status (single, married, separated, divorced or widowed)
- Time since diagnosis of AD (years and months);
- Cognitive impairment [MMSE and The Global Deterioration Scale (GDS)]^[68,78]^;
- Smoking (yes or no);
- Falls in the last year (number);
- Pharmacological treatment (name and number of pills per day);
- Comorbidities (name).

#### 2.7.1 Primary outcome variables

The primary outcome measures are the change in gait, balance, and bone health status during the study follow-up.

Physical functions such as gait and balance will be assessed by the POMA-T and the TUG. Both of them are widely and easily used tools applied in clinical settings with older adults and AD patients.^[16,30]^ And bone health status will be assessed by quantitative ultrasound (QUS).^[79–81]^

- POMA-T: We will perform the original POMA-T 28-point version. It consists of a balance scale [Balance performance oriented mobility assessment (POMA-B)] and a gait scale [Gait performance oriented mobility assessment (POMA-G)]. POMA-B can score 16 points (sitting balance, get up and sit up from a chair, immediate standing balance in the first 3 to 5 seconds, standing balance, balance with eyes closed and turning balance 360°). POMA-G can score 12 points (initiation of gait, step height, step length, step symmetry, step continuity, path deviation, trunk stability, and walk stance). Less than 19 points means high risk of falls, between 19 and 24 means risk of falls, and between 24 and 28 means no disturbance in gait or balance. Therefore, the POMA-T's cutoff point that predicts moderate or high risk of falling and disturbance in balance and gait was 24.^[16,17,22,82,83]^
- TUG: Is an easy tool to evaluate the gait and balance. It measures the time in seconds for the subjects to get up from a standard armchair, walk 3 m, turn, walk back to the chair and sit down. The cut-off point for normal mobility is 12 seconds and values > 30 seconds indicate a high level of dependence,^[30,84,85]^ although other authors are somewhat stricter with the interpretation of the TUG, in such a way that with a time <10 seconds, it is considered normal; <20 seconds mobility difficulties and low or moderate risk of falls; > 20 mobility problems with need of help and high risk of falls.^[84]^
- Calcaneal QUS: The bone health status will be assessed by ultrasound bone densitometry/sonometry. It is a safe, radiation-free modality that provides precise quantitative assessment of skeletal status, useful information in elderly for identifying patients at risk of developing osteoporosis and for assessing their risk of future fracture.^[79,80,86]^ Bone mass we will measured at the calcaneus (95% of trabecular bone) by using QUS (Sahara Hologic Clinical Bone Sonometer; Hologic lnc., Waltham, MA). QUS parameters will be measured, but we consider the estimated BMD (g/cm^2^) and T-score (comparison of the average mineral density of the patient's bone with a healthy young people) as primary outcome, because some researchers suggested that these parameters are more useful in the determination of subjects with low bone health status.^[79–81,86]^

#### 2.7.2 Secondary outcome variables

The secondary outcome measures are the change in other gait and balance tests, other bone health status parameters, and the nutritional status during the study follow-up.

- OLB (s): It assesses the ability of the patients to remain upright on 1 leg without support for at least 5 seconds. The test was performed by asking the participant to stand unassisted on 1 leg as long as possible (eyes open, barefoot, using whichever leg was spontaneously chosen by the participant), and each leg was tested. A shorter duration is considered a failure and is associated with a 2-fold increase in the risk of experiencing injurious falls.^[30,32,87]^ In addition, an abnormal OLB is a marker of more advanced dementia (worst baseline characteristic) and an independent predictor of cognitive decline in AD, and the OLB test could be adopted in clinical practice to identify AD patients at high risk of rapid cognitive decline.^[30,32]^
- FR (cm): It is a reliable and valid measure of proactive balance and is a sensitive measure strongly connected to physical frailty.^[34,88]^ FR is a measure of the distance in centimeters that the standing participant is able to reach forward from an initial upright posture to the maximal anterior leaning posture without moving or lifting the feet. The test will be performed 3 times, and the mean of the 3 attempts will be reported. For older adults, the subjects with a score between 15.2 and 25.4 cm are twice as likely to fall over than subjects with a score of 25.5 cm or more. And s34to older adults who reached 25.5 cm or more on this test.^[34]^
- Falls during the study: The number of falls of each subject during the study will be counted. Besides, we will use the World Health Organization questionnaire about older adult falls,^[89]^ which assesses aspects related to the history of falls. This questionnaire does not provide a numeric score.
- Other QUS parameters.^[80]^

Broadband ultrasound attenuation (BUA) (dB/MHz): It is the attenuation of broadband ultrasound when crossing the calcaneus from the transducer of the emitter to the transducer of the receiver with the densitometer. It is an indicator to determine the BMD.

Speed of sound (SOS) (m/s): It is the speed of the ultrasound conduction signal when crossing the calcaneus from the transducer of the emitter to the transducer of the receiver with the densitometer. It is an indicator to determine the BMD.

Quantitative ultrasound index (QUI) or stiffness index (SI) was calculated from BUA and SOS and expressed as absolute values. QUI is obtained by using the formula: QUI = (0.41x SOS) + (0.41x BUA)- 571.

- Nutritional status: Mini Nutritional Assessment (MNA) to assess the nutritional status and the relationship with cognitive stage, bone status, and physical functions. The MNA consists of 18 questions, with the total MNA score ranging from 0 to 30, where higher score indicating a better nutritional status.^[90]^ The total score was used to classify each patient into well nourished (MNA score >23.5), at risk of malnutrition (MNA score = 17.0–23.5), or malnourished (MNA score <17). MNA has a high sensitivity, specificity, and reliability.

### 2.8 Sample size calculation

The sample size calculation has been made based on the potential modification of the score of the POMA-T. For this, we have considered the results obtained in the previous pilot study with 9 patients in a 1-month follow-up, and the preliminary trial with 72 participants with a ratio of 3:1 to the intervention group and the control one during 6 months. In the pilot study, the POMA-T was modified by 1.7 points. Then, in the preliminary trial, we accepted to calculate the sample size, as statistically significant, with a difference greater than or equal to 1.7 points with a dropTout rate of 15%.

In our current study, the sample size has been estimated to conduct a randomized controlled clinical trial with a 1:1 ratio between the 2 groups. In this new calculation, considering the data from the pilot study and some of the available data from the preliminary trial, we have reduced the minimal detectable change in the POMA-T to 1.5 points, with a standard deviation of 2 points and we have increased the drop-out estimated rate during the study to 33% by the difficulty to avoid intervention biases in the control group fundamentally. Therefore, with this, and accepting an alpha risk of 0.05 and a beta risk of 0.2 in a 2-sided test, 84 subjects are necessary, 42 in each group. The sample size calculation was made with the software “GRANMO sample size and power calculator” (7.12 version).

### 2.9 Statistical analysis

Data will be analyzed by using the IBM-SPSS software package (version 23.0). Descriptive data analysis will be reported by groups as means ± standard deviation for quantitative variables and as frequencies and percentages for qualitative variables. In the graphical representation, bar graphs by groups will be used for quantitative variables, and pie charts to represent categorical variables. The main change from baseline to 6 months will be calculated by using intention-to-treat analysis for each outcome measure.

Initially, for comparisons between groups at baseline, *t* tests or Mann–Whitney *U* tests will be used for continuous variables, depending on normality, which will be checked for each using the Kolmogorov–Smirnov test and normal probability box-plots, and the Chi-square test or Fisher test will be used for quantitative variables.

To analyze the efficacy of the multidomain intervention on the outcome variables, both in the primary and secondary outcomes, multivariate analysis of variance (MANOVA) will be applied with repeated measures (4 levels) to assess interactions between the factors and differences by categorical variables, with covariates, and pairwise comparisons post-hoc to determine whether there are significant differences between groups and within groups. The effect size for the MANOVA model will be calculated by the partial eta square (η^2^) that shows how much variance is explained by the independent variable.

We will carry out correlations with Pearson *r* coefficient to determine whether the values of 2 or more quantitative variables change in conjunction or are related in some of the moment of the study (time-factor in the statistical model).

For the analysis of the falls data, we will calculate the incidence rate during the study and the prevalence proportion by group and by sex at the end of the study (point prevalence). Subsequently, we will calculate the relative risk to estimate the magnitude of the association between the participation to the MPEP and falls, and indicate the likelihood of fall in the intervention group relative to the control group and also by sex.^[91]^

The statistical analysis will be conducted at a 95% confidence level. A *P* value < .05 will be considered to indicate statistical significance in all analyses.

## 3 Discussion

This study is part of a line of research and has been planned in the context of physical exercise as a nonpharmacological treatment, along with other interventions such as diet, in elderly and patients with cognitive impairment, considering them as vulnerable or frail population. There are many studies that are showing the beneficial effects of physical exercise on the changes of aging in elderly^[52,54,57,70]^ and in patients with cognitive decline.^[3,24,30,31,35,36,40,56,58]^ Patients with AD are a growing population group, with disturbing epidemiological data for a near future with important consequences from the health, social, and economic points of view.^[4,5]^

For these reasons, in this study, we want to evaluate the short and medium-term effects of a MPEP associated with a MeDi in people with AD on aspects that can improve their quality of life and their degree of autonomy. Taking into account the frailty of this population and the comorbidities correlated to cognitive decline and aging, we will evaluate a large number of outcome variables to assess the impact of the proposed intervention program on physical functions and bone health status with the intention of reducing the risk of falls and its serious consequences, mainly fractures. In such a way, that this MPEP with a MeDi has several specific objectives that attempt to avoid, delay, and/or minimize some of the frail conditions of these patients.

In our study, we want to contribute to the evidence on the reduction of bone fractures and their consequences. To do this, we plan to reduce the risk of fractures, intervening to decrease bone mass loss and the risk of falls as the main causal factors of fractures in older people and, even more, in people with cognitive impairment, such as patients with AD. There are studies that have addressed these aspects separately, but there are no studies that have worked on them together, and we will also look for associations or correlations between them. For this reason, we also will create a registry of falls to see the preventive effect of the intervention, as well as the relationships of each outcome variable with falls. During the 6 months of the study, changes in BMD, gait and balance, and nutritional status will be monitored and analyzed.

In recent years, physical activity has increased exponentially in community-dwelling and institutionalized older people, as well as in different risk populations such as patients with AD. However, there is no consensus in the evaluation instruments and in the interventions. We must have, as a fundamental objective to apply, the results achieved through daily clinical practice and, consequently, we have to be aware of the difficulties and the great variability of situations that these patients have due to many and varied factors. Therefore, it is important to be able to standardize and simplify evaluation protocols, with valid, reliable, low-cost tools that are easy to apply in clinical practice, as well as intervention protocols capable of being included in the multidisciplinary groups of specialized centers for people with AD, and that the protocols can be adapted to other scenarios, such as work at home. If the intervention of this present study appears effective, one of its advantages is its applicability in the health care system. It is necessary to support the visibility of the evidence of the effects of physical exercise on all the factors associated with AD, and its comorbidities, to favor an increase of investment from both public and private clinic care. Implementation of research into clinical practice is of importance for the overall quality in health research.^[92]^

Our study has been planned with a well-coordinated multidisciplinary group, will make it easier to monitor some factors that are normally difficult to control in the clinic and in research, such as a correct diagnosis, medication, or diet. In addition, we hope to maintain a very high adherence to the intervention in the participants of the Center, among other things, by the intervention in small groups of 5 to 8 people. In this way, the values of group work will be favored, such as group feelings, stimulates, improves self-esteem, and generates more adherence than individual strategy,^[64,65]^ and at the same time, this way will allow the necessary individualization for corrections and adaptations to each patient.

### 3.1 Strengths and limitations

We consider having some strengths points in our study. The design of this study has been based on the experience and results of previous pilot study and preliminary nonrandomized controlled clinical trial. The procedures will be carefully conducted by a multidisciplinary professional group highly qualified in the care and treatment of patients with AD. The novelty of our study is based on the fact that it is the first longitudinal clinical trial aimed at assessing the effects of a MPEP with a MeDi in short- and medium-term over 6 months on the bone health status and the risk of falls by correlating them with gait and balance disorders.

Some limitations of this study should be noted. General conclusions can only be drawn with caution and no conclusions about our intervention results should be drawn beyond institutionalized patients with AD, which suggests that they are more affected. Finding eligible elderly people with AD, who do not attend a center where they receive some type of intervention with physical exercise, is currently difficult, which makes intervention biases easily can appear. This can considerably increase drop-out rate during follow-up and the length of the recruitment period. Other studies should attempt to address strategies to increase the recruitment capacity of eligible patients for the control group, and to carry out experimental designs with 2 or more intervention arms.

In our future studies, in the same line of research, we also want to assess in greater depth the impact of diet combined with exercise on our outcome variables and analyze the influence of the nutritional status of patients with cognitive impairment, as well as their relationships with physical, cognitive, and bone health. And the study results will be released to the participating physicians, referring physicians, patients, and the general medical community.

## Acknowledgments

The authors would like to thank all the participants and families for their cooperation in the study.

## Author contributions

ASP-G, MCS-S, and RM-S designed the study. ASP-G, FJB-I, and RM-S were involved in designing the multicomponent exercise program as well as outcome measures. FS-G, JEH-X, and FJB-I provided methodological advice for the design of the study. JEH-X and RM-S made the design of the statistical analysis. ASP-G and RM-S wrote the first draft of this manuscript. All authors read, provided critical revisions, and approved the final manuscript for publication.

**Conceptualization:** Ana Silvia Puente-González, María Carmen Sánchez-Sánchez, Fausto José Barbero-Iglesias, Roberto Méndez-Sánchez.

**Formal analysis:** Juan Elicio Hernández-Xumet, Roberto Méndez-Sánchez.

**Methodology:** Ana Silvia Puente-González, Felipe Sánchez-González, Juan Elicio Hernández-Xumet, María Carmen Sánchez-Sánchez, Fausto José Barbero-Iglesias, Roberto Méndez-Sánchez.

**Writing – original draft:** Ana Silvia Puente-González, Roberto Méndez-Sánchez.

**Writing – review & editing:** Ana Silvia Puente-González, Felipe Sánchez-González, Juan Elicio Hernández-Xumet, María Carmen Sánchez-Sánchez, Fausto José Barbero-Iglesias, Roberto Méndez-Sánchez.

## Abbreviations

Abbreviations: AD = Alzheimer disease, BMD = bone mineral density, BMI = body mass index, BUA = broadband ultrasound attenuation, Center-AD = Center for people with Alzheimer's Disease and other Dementias, CONSORT = Consolidated Standards of Reporting Trials, FR = functional reach test, GDS = Global Deterioration Scale, MANOVA = multivariate analysis of variance, MeDi = Mediterranean diet, MMSE = Mini-mental state examination, MNA = Mini Nutritional Assessment, MPEP = Multicomponent physical exercise program, OLB = 1-leg balance test, POMA-B = Balance performance oriented mobility assessment, POMA-G = Gait performance oriented mobility assessment, POMA-T = Tinetti performance oriented mobility assessment, QUI = quantitative ultrasound index, QUS = quantitative ultrasound, SI = stiffness index, SOS = speed of sound, SPIRIT = Standard Protocol Recommendations for Interventional Trials, TUG = timed up and go test.
